# Photosynthetic Performance and Yield Losses of Winter Rapeseed (*Brassica napus* L. var. *napus*) Caused by Simulated Hail

**DOI:** 10.3390/plants13131785

**Published:** 2024-06-27

**Authors:** Piotr Dąbrowski, Łukasz Jełowicki, Zuzanna M. Jaszczuk, Olena Kryvoviaz, Hazem M. Kalaji

**Affiliations:** 1 Department of Environmental Management, Institute of Environmental Engineering, Warsaw University of Life Sciences—SGGW, Nowoursynowska St. 159, 02-787 Warsaw, Poland; 2 OPEGIEKA Sp. z o.o., Aleja Tysiąclecia 11, 82-300 Elbląg, Poland; lukasz.jelowicki@opegieka.pl; 3 Faculty of Agriculture and Ecology, Warsaw University of Life Sciences—SGGW, Nowoursynowska St. 159, 02-787 Warsaw, Poland; 4 Department of Plant Physiology, Institute of Biology, Warsaw University of Life Sciences—SGGW, Nowoursynowska St. 159, 02-787 Warsaw, Poland

**Keywords:** abiotic stresses, bioindicator, chlorophyll *a* fluorescence, gas exchange, pulse-modulated amplitude fluorescence, JIP-test

## Abstract

Winter oilseed rape (*Brassica napus* L.), Europe’s foremost oilseed crop, is significantly impacted by hailstorms, leading to substantial yield reductions that are difficult to predict and measure using conventional methods. This research aimed to assess the effectiveness of photosynthetic efficiency analysis for predicting yield loss in winter rapeseed subjected to hail exposure. The aim was to pinpoint the chlorophyll fluorescence parameters most affected by hail stress and identify those that could act as non-invasive biomarkers of yield loss. The study was conducted in partially controlled conditions (greenhouse). Stress was induced in the plants by firing plastic balls with a 6 mm diameter at them using a pneumatic device, which launched the projectiles at speeds of several tens of meters per second. Measurements of both continuous-excitation and pulse-modulated-amplitude chlorophyll fluorescence were engaged to highlight the sensitivity of the induction curve and related parameters to hail stress. Our research uncovered that some parameters such as F_s_, F_m’_, Φ_PSII_, ETR, F_o_, F_v_/F_m_, and F_v_/F_o_ measured eight days after the application of stress had a strong correlation with final yield, thus laying the groundwork for the creation of new practical protocols in agriculture and the insurance industry to accurately forecast damage to rapeseed crops due to hail stress.

## 1. Introduction

Winter rapeseed (*Brassica napus* L., Brassicaceae) stands as a pivotal crop within cereal-dominant crop rotations across European agricultural landscapes. It is prized for its seeds, which are processed into a versatile oil catering to human nutrition, industrial applications, and biodiesel production. Additionally, its by-products serve as valuable animal feed [1]. Among biofuel sources, it boasts the highest energy potential for biodiesel in Europe, particularly the canola variety known for its low erucic acid and glucosinolate content [2].

In recent decades, the escalation of adverse weather events, alongside consequent economic losses, has become more apparent. The agricultural sector’s vulnerability to climate change is increasingly recognized [3]. The World Meteorological Organization (WMO) described hail as a solid form of precipitation, varying from transparent to opaque and composed of ice particles [4]. These can take spherical, conical, or irregular shapes, typically measuring 5 to 50 mm in diameter. Hail formation is influenced by surface conditions and atmospheric circulation, with its development spurred by localized surface features that promote thermal convection and air turbulence during the passage of cold fronts over superheated surfaces due to specific meteorological conditions [4]. The extent of plant damage from hail depends on various factors including hail size, storm duration, wind speed, crop type, plant growth stage, maturity level, and remaining harvest time [5]. June and July mark the peak hail frequency in Europe, aligning with critical growth stages (BBCH 59 to 69) in crops like rapeseed, where blooms are formed or pod formation is underway [6], making them highly susceptible to mechanical stress and potential agricultural productivity losses. Additionally, the annual frequency of hail events has risen due to climatic changes [4]. Farmers are advised to secure agricultural insurance policies to safeguard against economic losses. Assessing damages after hailstorms involves field investigations by insurance adjusters, using interpretation of photo and professional judgment to identify hail damage thresholds across plant species. Accurate, rapid prediction of yield loss post-hailstorm is vital for setting insurance compensation standards and guiding farmers in post-damage management [7,8].

It is important to note that physiological changes in plants due to hail precede morphological changes, with photosynthesis being particularly vulnerable to hail-induced damage [7]. Photosynthetic activity, especially within Photosystem II (PSII)—the most stress-sensitive component of the photosynthetic machinery—plays a critical role in the plant’s response to environmental stressors. Chlorophyll fluorescence (ChFl) kinetics analysis emerges as a potent tool for gauging the impact of stress on photosynthesis, offering a reliable assessment of photochemical efficiency. This analysis is crucial for understanding the dynamics of the electron transfer process in photosynthesis, shedding light on the effects of environmental stress on photosynthetic performance [9,10]. There are two main techniques for measuring ChFl signals: continuous-excitation chlorophyll fluorescence and pulse-modulated amplitude (PAM) fluorescence. The first technique necessitates a preliminary dark adaptation period of about 20 min for the leaf before measurement can be taken. The JIP-test, a widely used analytical procedure developed by Strasser et al. in 2004 [11], assesses the functionality of Photosystem II (PSII) and its sensitivity to various stresses. Conversely, the PAM fluorescence method does not require a preliminary dark adaptation of the leaf samples because it uses a modulated light source to induce chlorophyll fluorescence. The actinic light is toggled on and off at programmed frequencies, allowing for the measurement of variable components of the induced fluorescence. This facilitates a comprehensive analysis of the dynamic changes in chlorophyll fluorescence. A major advantage of this method is its ability to conduct measurements in natural sunlight, significantly enhancing the applicability of PAM fluorescence for evaluating photosynthetic activity in authentic environmental conditions, as noted by [12].

To address the gap in the literature regarding the estimation of yield losses in winter rapeseed due to hail, there is a notable absence of comprehensive data on utilizing parameters measured through both continuous-excitation and pulse-amplitude modulated (PAM) fluorescence techniques simultaneously. Consequently, this study aimed to pinpoint the chlorophyll fluorescence (ChFl) characteristics most vulnerable to hail damage, proposing their use as bioindicators for predicting yield losses in winter rapeseed exposed to hail stress. Identifying these key variables enables the study to achieve two main objectives: first, to elucidate the response of the photosynthetic apparatus in rapeseed to hail stress, thereby providing insights into the crop’s adaptability and resilience under such adverse conditions, and second, to underscore significant chlorophyll fluorescence parameters that could aid in the prediction of yield losses, offering a novel approach to assessing the impact of hail on crop productivity.

## 2. Results

### 2.1. Yield Loses

The yield of winter oilseed rape was influenced by hail stress (Table 1). Under control conditions, the yield was 3.84 t ha^−1^. Exposure to hail led to a significant decrease in yield by 53.9%.

### 2.2. Gas Exchange and Chlorophyll Content Index Results

It was observed that all of the measured parameters were sensitive to hail stress (Table 2). CO_2_ assimilation (*A_n_*) in a non-stressed plant was 32.2 µmol CO_2_ m^−2^ s^−1^. Hail caused a reduction by 32.2%. Stomatal conductance (*g_s_*) in non-stressed plants was 0.34 µmol CO_2_ m^−2^ s^−1^, and it was reduced by hail by 47.1%. Substomatal CO_2_ concentration in non-stressed plants was 238 (µmol CO_2_ mol^−1^), and hail caused an increase in its values by 47.5%. Transpiration rate (*E*) in the control plant was 17.1 mmol H_2_O m^−2^ s^−1^ and was reduced by hail by 48%. Chlorophyll content index was reduced by hail by 36.4% in comparison to the control.

### 2.3. JIP-Test Measurements

**Figure 1:**
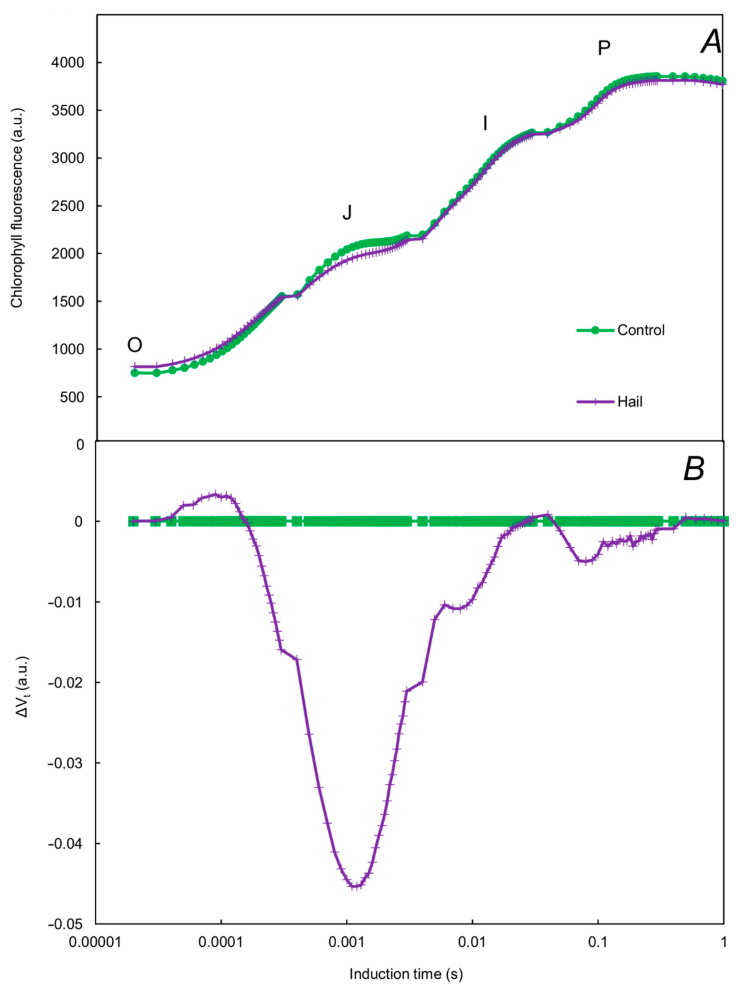
Induction curves of chlorophyll *a* fluorescence (**A**) and differential curves of ΔV_t_ (obtained by subtracting the control curve from the first sample) (**B**) of winter oilseed rape under the influence of hail.

The course of the OJIP curve was significantly influenced by hail stress (Figure 1). At the J point of the curve, a decrease was observed in comparison to the control. After the normalization procedure, changes were also evident at point O (increase in ChFl value compared to the control) and at point P (decrease in ChFl value compared to the control).

**Figure 2:**
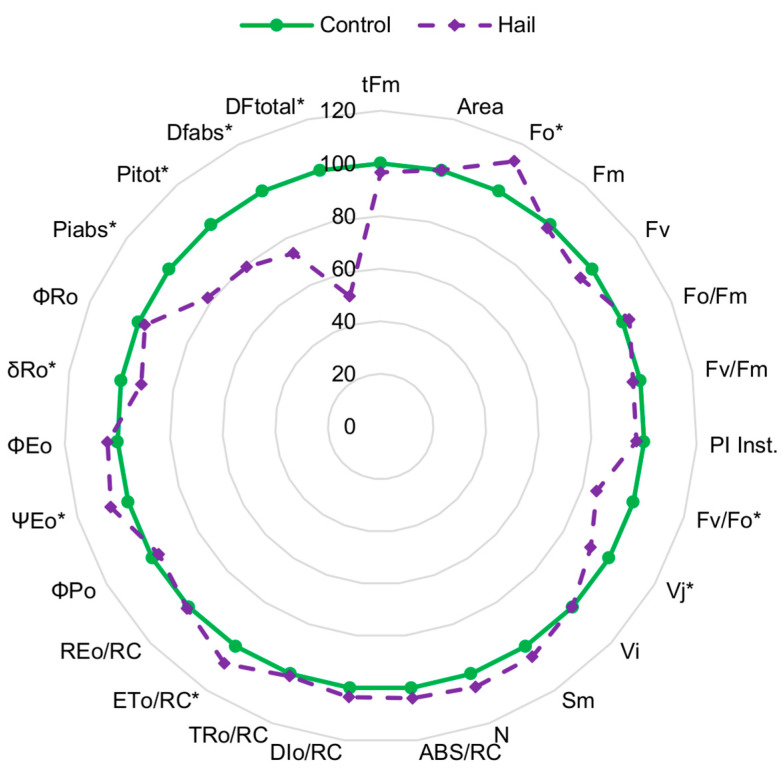
The JIP test parameters normalized to the values before stress application (control) as radar plots of winter rapeseed under the influence of hail (a.u. ± S.D.). Means within particular parameters marked by the asterisk differed significantly from the control (*p* < 0.05, *n* = 16). Time to reach the maximal fluorescence (t_Fm_), area above the OJIP curve (Area), minimal fluorescence (F_o_), maximal fluorescence (F_m_), variable fluorescence (F_v_), ratio of the efficiency of primary photochemical reactions (F_o_/F_m_), maximum quantum yield of primary photochemical reactions (F_v_/F_m_), maximum efficiency of the water diffusion reaction (F_v_/F_o_), relative variable fluorescence at the J and I steps (V_j_ and V_i_), normalized total area above the OJIP curve (S_m_), absorption flux per one active RC (ABS/RC), energy flux not intercepted by an RC (DI_o_/RC), energy flux trapped by one active RC (TR_o_/RC), rate of electron transport by one active RC (ET_o_/RC), efficiency index expressed as the density of RCs per chlorophyll (Chl) (RE_o_/RC), maximum quantum yield of primary photochemical reactions (ϕ_Po_), quantum yield for electron transport (ϕ_Eo_), efficiency/probability that an electron moves further than Q_A_^−^ (ψ_Eo_), efficiency/probability with which an electron from the intersystem electron carriers is transferred to reduce end electron acceptors at the PSI acceptor side (δ_Ro_), quantum yield for reduction of end electron acceptors at the PSI acceptor side (ϕ_Ro_), performance indexes (PI_inst_, PI_abs_, and PI_tot_), driving forces (DF_abs_ and DF_tot_).

Some parameters of the JIP test were sensitive to hail stress (Figure 2). Among the parameters that significantly increased their values under the influence of stress were minimal fluorescence (F_o_), electron transport flux per RC (ET_o_/RC), efficiency/probability that an electron moves further than Q_A_^−^ (ψ_Eo_), and efficiency/probability with which an electron from the intersystem electron carriers is transferred to reduce end electron acceptors at the PSI acceptor side (δ_Ro_). On the other hand, among the parameters that significantly decreased their values were F_v_/F_o_, V_J_, performance indexes (PI_abs_ and PI_tot_), and relative driving forces (DF_abs_ and DF_tot_).

### 2.4. PAM Measurements

**Figure 3:**
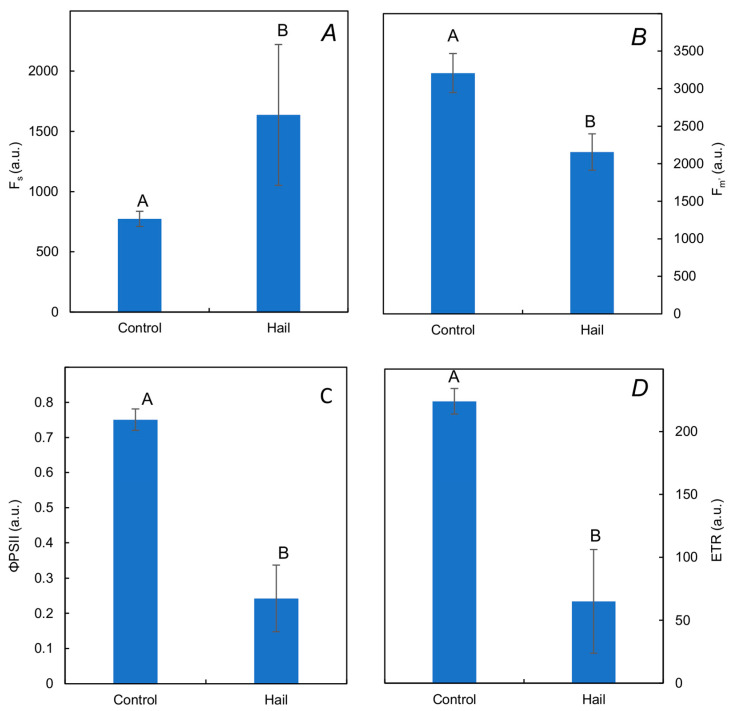
The PAM (pulse-modulated amplitude fluorescence) parameters: steady-state fluorescence at any light level (F_s_) (**A**), maximum fluorescence from light-adapted leaf (F_m’_) (**B**), estimated effective quantum yield (efficiency) of PSII photochemistry at given PAR (Φ_PSII_) (**C**), and electron transport rate (ETR) (**D**) of winter rapeseed under the influence of hail: (t ha^−1^ ± S.D.). The means marked by the same letter did not differ significantly (*p* < 0.05, *n* = 16).

All parameters obtained from PAM measurements were significantly influenced by hail stress (Figure 3). Under control conditions, the steady-state fluorescence (F_s_) parameter was 773 (a.u.). Exposure to hail led to a significant increase in its value by 112%. Under control conditions, the maximum chlorophyll *a* fluorescence in light-adapted leaves (F_m’_ parameter) was 3208 (a.u.), and statistical analysis did confirm significant influence of hail on this parameter. Exposure to this stress caused reduction in this parameter by 32.8%. Under control conditions, the estimated effective quantum yield (efficiency) of PSII photochemistry at given PAR (Φ_PSII_ parameter) was 0.75 (a.u.), and statistical analysis did also confirm the influence of hail on this parameter. Exposure to this stress caused a reduction in this parameter in comparison to the control by 67.7%. The electron transport rate (ETR parameter) also underwent reduction by 71.0% in comparison to the control.

### 2.5. Relationship between Hail Stress and Individual Parameters of Photosynthetic Performance

A significant correlation was observed between yield loss and all measured gas exchange parameters (Table 3). Stomatal CO_2_ concentration was correlated negatively, and all other parameters (*A_n_*, *g_s_* and *E*) were correlated positively. Also, chlorophyll content index (CCI) was correlated positively.

It was discovered that some specific chlorophyll *a* fluorescence parameters were significantly correlated with the yield loss attributed to hail stress, as detailed in Table 3 (parameters marked by asterisk). Among the parameters positively correlated were F_m’_, Φ_PSII_, ETR, F_v_, F_v_/F_m_, F_v_/F_o_, V_J_, ϕ_Po_, PI_abs_, PI_tot_, DF_abs_, and DF_total_. The values of correlation coefficients of these parameters ranged from 0.38 to 0.95. Among the parameters negatively correlated were F_s_, ET_o_/RC, ψ_Eo_, and O point from the OJIP curve. The values of correlation coefficient of these parameters ranged from −0.49 to −0.77.

**Figure 4:**
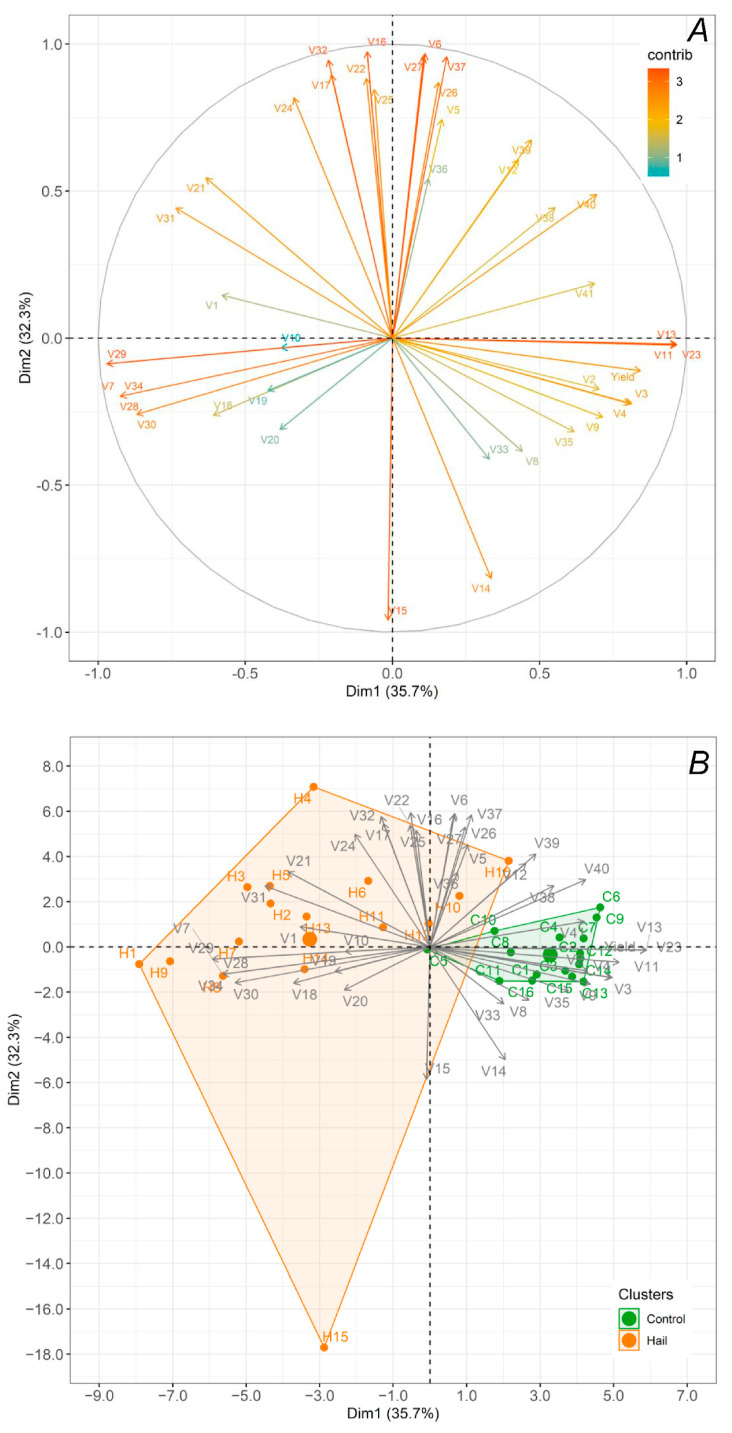
Principal component analysis of the salt stress effect on the photosynthetic machinery of winter oilseed rape and its yield under hail stress: vector graphs showing the relative “contribution” of each input variable to the formation of the principal components (**A**); biplot (**B**). CCI (1), *A_n_* (2), *g_s_* (3), *C_i_* (4), *E* (5) F_s_ (6), F_m’_ (7), Φ_PSII_ (8), ETR (9), t_Fm_ (10), Area (11), F_o_ (12), F_m_ (13), F_v_ (14), F_o_/F_m_ (15), F_v_/F_m_ (16), PIinst. (17), F_v_/F_o_ (18), V_j_ (19), V_i_ (20), S_m_ (21), N (22), ABS/RC (23), DI_o_/RC (24), TR_o_/RC (25), ET_o_/RC (26), RE_o_/RC (27), ϕ_Po_ (28), ϕ_Eo_ (29), ψ_Eo_ (30), δ_Ro_ (31), ϕ_Ro_ (32), PI_abs_ (33), PI_tot_ (34), DF_abs_ (35), DF_tot_ (36), O point (37), K point (38), J point (39), I point (40), and P point (41).

In the vector graphs (Figure 4A), the relative “contribution” of each input variable to the formation of the principal components (Dim1 and Dim2) is presented. The magnitude of the vector is an indicator of the hail influence on corresponding ChFl parameter, and the vector direction depends on this impact on the Dim1 and Dim2 value. The modifications in the first principal component (Dim1) determined 35.7% of total changes, and the second component Dim2 reflected 32.3%. The analyzed ChFl parameters had different sensitivity to hail stress, as well as different contributions in the formation of principal components. There were groups of parameters, which were positively correlated with yield, such as *A_n_*, *g_s_*, *C_i_*, ETR, Area, F_m_, and ABS/RC. On the other hand, there were parameters negatively correlated with yield, such as F_m’_, ϕ_Po_, ϕ_Eo_, and ψ_Eo_. It is worth noting that there was the largest group of parameters, for which vectors had 90° to yield vectors. This group can be classified with parameters like *E*, F_s_, F_v_/F_m_, N, DI_o_/RC, ET_o_/RC, TR_o_/RC, RE_o_/RC, and O point.

The majority group of the control and stressed plants of winter rapeseed are presented (Figure 4B). For plants not subjected to hail stress, the values of the principal components varied at around 0 to 5 for Dim1 and −1 to 2 for Dim2. In plants subjected to hail stress, incubation of the physiological state of the photosynthetic apparatus was changed—the population varied at around −8 to 5 for Dim1 and around −18 to 7 for Dim2.

## 3. Discussion

Literature reports on the impact of hail on plant physiology are limited. Existing studies indicate that photosynthetic efficiency, assessed through gas exchange measurements and the analysis of specific chlorophyll fluorescence (ChFl) parameters, significantly decreases in trees [13,14] and shrubs [15] subjected to this type of mechanical stress. Additionally, a decline in yield was observed in these studies, as well as in potatoes [16].

The primary objective of our research was to investigate whether particular physiological parameters, measured through gas exchange and both continuous-excitation and pulse-amplitude-modulated (PAM) chlorophyll fluorescence techniques, could serve as reliable indicators of hail stress in winter oilseed rape plants. We posited that ChFl measurements might provide a dependable method for detecting hail stress in oilseed rape prior to the manifestation of visible symptoms. This hypothesis is founded on the presumed correlation between ChFl signals and photosynthetic efficiency.

The primary energy source for all autotrophic plants’ growth and development is photosynthesis in their leaves. It is now commonly acknowledged that stomatal conductance and/or leaf biochemical capacity are the primary factors limiting photosynthesis in C_3_ plants under stress [17]. Our studies confirmed such gas exchange parameters as CO_2_ assimilation and H_2_O transpiration and stomatal conductance decreased in winter oilseed rape under hail stress. Meanwhile, intercellular CO_2_ concentration increased. These results can suggest that CO_2_ accumulates within intercellular spaces of the leaf when it is not assimilated by Rubisco [18].

The pivotal discovery of our research was the identification of specific points along the OJIP curve that are sensitive to hail stress. Our findings revealed that the J point of the curve maintained its sensitivity under hail stress conditions. Additionally, the O and P points also showed some sensitivity, as indicated by their values being comparable after subtracting the control curve from the first sample (ΔV_t_). Notably, there is a lack of information in existing literature regarding alterations in the shape of the OJIP curve in response to hail stress. Nonetheless, the pattern of sensitivity across the OJIP curve points in plants subjected to various abiotic stresses has been extensively documented [19,20]. Furthermore, modifications in the values of specific points on the curve were also observed in rapeseed plants experiencing temperature stress [21,22], underscoring the potential of these markers in assessing plant response to environmental pressures.

Next, the specific JIP-test parameters were analyzed and presented in a radar plot format. It was proved that only a few parameters were receptive to hail stress. This emphasizes that mechanical stress has a complex effect on the physiological status of winter oilseed rape and highlights how important it is to use modern fluorescence techniques to identify and measure stress responses. This phenomenon can be followed well based on the values of the performance index parameters (PI_abs_ and PI_tot_) and driving forces (DF_abs_ and DF_tot_), which were significantly changed by this stress. The sensitivity of those parameters on low-temperature stresses in winter oil seed rape was confirmed by [21,22,23]. The sensitivity of those parameters was also confirmed in other plant species under different abiotic stresses [24,25]. On the other hand, the F_v_/F_m_ parameter was significantly decreased by simulated hail in apple trees [14] and in grapes [26]. Our research did not confirm a significant decrease in the F_v_/F_m_ parameter in winter oil rapeseed under hail stress; however, there was a significant correlation between its values and yield. Moreover, as in the studies of [14,26], we confirmed the increase in the F_o_ parameter caused by this stress.

Hail stress caused significant modifications in all four PAM parameters measured in light-adapted samples illuminated with actinic light. These parameters are important for understanding the molecular dynamics of photosynthesis under various biotic and abiotic stresses [9,27]. Many researchers are certain that PAM analysis is more intricate than OJIP analysis since it integrates both photochemical and non-photochemical quenching [28,29].

Our research proved that F_s_ (a parameter also known as steady-state fluorescence, which reflects the relative fluorescence intensity) is sensitive to hail stress. The increase in this parameter can be interpreted as an imbalance between the rates of ATP, NADPH synthesis, and CO_2_ fixation [12]. In such a situation, the Calvin–Benson cycle devours less ATP and NADPH for CO_2_ fixation than is generated by the primary processes of photochemical photosynthesis [30]. As a consequence, the quantum efficiency of PSII photochemistry is reduced. The increase in this parameter can also indicate that some PSII reaction centers are unable to utilize excitation energy effectively under light conditions, which led to an increase in non-photochemical de-excitation (NPQ) [31].

The F_m’_ parameter was significantly decreased by hail stress. Moreover, there was a significant correlation between its values and the yield of winter oil rapeseeds. In general, fluctuation of this parameter reflects changes in the rate constant of regulated non-photochemical quenching. The changes in its values might express the extent of the regulated loss of non-photochemical energy [32].

The decrease in the Φ_PSII_ parameter in winter oil rapeseed under hail was noted in our research. It should be underlined that our research confirms the highest relationship of this parameter (among all tested ChFl parameters) and yield loss. The correlation coefficient (*r*) between these parameters was 0.94. This parameter represents the fraction of the light energy absorbed by PSII, which drives photosynthetic electron transport [12]. It is frequently used in field research and might be interpreted as the effective quantum yield of the PSII photochemistry related to the actual fraction of photochemically active PSII RCs (qP) [33].

ETR represents the rate of photosynthetic processes in plant samples and correlates well with the quantum yield of CO_2_ assimilation and stomatal conductance [12]. The decrease in the ETR parameter in winter oil rapeseed under hail was noted in our research. This phenomenon was found in apple trees [14] and in grapes [28].

A multi-parametric analysis was used to evaluate the stress effects in winter rapeseed in order to identify parameters that are most sensitive to the plant stress response. The separate parameters are not fully autonomous (both in the case of the JIP-test parameters and PAM parameters) because some of them are calculated on the basis of points of the chlorophyll fluorescence curve [34]. Some of them are connected by mathematical expressions (e.g., φ_Po_ and φ_Do_). Plant stress response is accurately described by each of the ChFl parameters, yet each parameter represents a different characteristic of photosynthetic machinery. Although the comparison of the samples was overly complicated, the multiparametric description based on the constellation of factors provides a more detailed explanation of plant photosynthesis. Principal component analysis (PCA), which enables a better understanding of the stressor effect on the photosynthetic machinery as a whole, is an efficient way to employ such a set of experimental characteristics [35]. By utilizing the PCA-based technique, we are able to fully utilize the fluorescence data and derive new features that accurately depict the response of plant photosynthetic processes to stress. The stress-induced changes in the plants under investigation were displayed in Figure 4 as a 2D graph on a plane with Cartesian coordinates labeled “Dim1” and “Dim2” for easier visualization.

Finally, we concluded that the calculation of crop production losses due to hail stress can be facilitated by employing measurements of plant photosynthetic efficiency to track changes in the winter oilseed rape. Our research revealed that some of chlorophyll fluorescence parameters such as F_s_, F_m’_, Φ_PSII_, ETR, F_o_, F_v_/F_m_, and F_v_/F_o_ measured directly after stress application have a strong correlation with final yield, thus laying the groundwork for the creation of new practical protocols in agriculture and insurance industry to accurately forecast damage to rapeseed crops due to hail stress. Our research improved our knowledge of how vulnerable crops are to the sudden hailstorms. However, this paper reports on a study that was limited to a single crop variety and a one-year experiment that was carried out in partially controlled conditions. Future studies should be conducted in the field on a larger number of crop varieties and experimental seasons to expand on these insights and support our findings. Moreover, our research did not set out to directly compare the performance of different algorithms in forecasting crop yield losses. Existing literature indicates that nonlinear algorithms, such as random forests, support vector machines, and artificial neural networks, might be more effective than linear algorithms in processing chlorophyll fluorescence data to predict yield losses.

## 4. Materials and Methods

### 4.1. Experimental Design and Plant Growth Conditions

The experiment was carried out in semi-controlled conditions in an open vegetation hall (open greenhouse) at Warsaw University of Life Sciences (WULS). Winter rapeseed plants (variety LG Areti, Limagrain, Saint Beauzire, France,) were sown in 32 pots (5 seeds per pot) on 12 September 2022. Each pot contained 6 kg of substrate composed of 60% peat, 30% composted bark, and 10% sand. After preparing the mixture, 11 kg m^−3^ of chalk was added (to reach pH 6), followed by MIS4 fertilizer + microelements (120 g m^−3^) (Intermag, Olkusz, Poland). On 2 October, 0.5% calcium nitrate + folium 0.6% + L amino H 0.6% was sprayed. On 5 October, 3% MgSO^4+^ Radiculum 0.6% was sprayed.

The outside section of the greenhouse was then used to house all of the pots, creating a natural environment. At this time, no new stressors were added. The most significant weather conditions for the experiment are listed in Table 4.

Plants that had been dormant during the winter broke their hibernation on 29 January 2023, when all of the pots were moved into a closed interior area of the greenhouse. During this time, the average daily temperature was about 18 °C, and HPSC lamps were used to provide additional lighting for the plants (200 umol m^−2^ s^−1^).

On 20 April 2023, the rape plants were in phase BBCH 65–67 (full flowering: 50% of the flowers on the main inflorescence are open, older petals fall off) [6]. At this time, pots were divided randomly into 2 groups (16 pots per each group). Each group of vases constituted a separate research variant. The variants are presented below:

(i) Control;
(ii) Hail stress.

Hail stress was simulated by firing at plants with a pneumatic device that launched 6 mm diameter plastic balls at speeds of several tens of meters per second. The firing was carried out from an elevated position (distance of a few meters) to mimic hail falling from above, ensuring each plant received an identical number of impacts. All measurements of photosynthetic efficiency were taken on 28 April 2023. Subsequently, the plants were grown under natural conditions in the open section of the greenhouse until the end of the vegetation period. The harvest was collected on 7 July 2023.

The measurements of chlorophyll fluorescence, greenness index, and gas exchange were always performed on the same part of the chosen leaf: halfway along its length and one-quarter of its width. Leaves were selected from the middle canopy layers. In the case of non-stressed plants (control), measurements were conducted on intact leaves. However, in the case of plants treated with hail, only damaged leaves were measured, avoiding close spots of the hail damage.

Stress was applied on 20 April, when the rape plants were in phase BBCH 65–67 (full flowering: 50% of the flowers on the main inflorescence are open, older petals fall off). This term was chosen because, at this growth stage, the occurrence of hail causes the greatest losses. The measurements were performed on 28 April, so 8 days after stress application.

### 4.2. Yield Measurements

Following the photosynthetic performance measurement, the plants underwent a natural desiccation process. Afterwards, seeds were harvested separately from each pot and weighed to determine the actual yield. The results obtained were then converted to t h^−1^.

### 4.3. Gas Exchange Measurements and Chlorophyll Content Index Measurement

One plant from each pot was selected at random. To improve the correlation index between gas exchange, chlorophyll content index, and ChFl signals, all the measurements were made on previously marked leaves. On each plant, one leaf was selected for the measurement. Measurements were always performed on the same part of the leaf: halfway along its length and one-quarter of its width. Leaves were selected from the middle tiers, specifically the fourth or fifth tier. The photosynthetic rate in terms of net CO_2_ assimilation (*A_n_*), stomatal conductance (*g_s_*), transpiration rate (*E*), and the substomatal CO_2_ concentration (*C_i_*) were all measured on this same leaves as ChFl and CCI using a portable gas analyzer Lcpro^+^ (ADC BioScientific Ltd., Hoddesdon, UK). This open-gas exchange system operated during measurement on a differential mode at a 150 mol s^−1^ flow rate of ambient air. The measurements were taken after the stabilization of conditions in the chamber.

The chlorophyll content index (CCI) measurements were made by using a CCM-200 (Opti-Sciences, Inc., Hudson, NH, USA) chlorophyll content meter.

### 4.4. Chlorophyll a Fluorescence Measurement

Chlorophyll a fluorescence was assessed using two fluorimeters:

- HandyPEA (Hansatech Instruments Ltd., King’s Lynn, UK);
- FMS-2 (Hansatech Instruments Ltd., King’s Lynn, UK).

To better visualize the influence of the stress on the dynamics of the chlorophyll transients, the relative variable fluorescence intensity (V_t_) was calculated. At the next stage, the differences of relative variable fluorescence intensity (ΔV_t_) were calculated by subtracting the normalized fluorescence values (between O and P steps) recorded in control plants and under stress. V_t_ and ΔV_t_ were calculated according to the formulas:

V_t_ = (F_t_ − F_o_)/(F_m_ − F_o_) (1)

ΔV_t_,_stress_ = V_t_,_stress_ − V_t,control_ (2)

All chlorophyll fluorescence parameters measured by HandyPEA after dark adaptation (JIP test) are presented in Table 5.

Before the measurements, the middle section of each leaf was dark adapted for at least 25 min by using Hansatech leaf clips. A brief non-actinic light flash was applied to the leaf just before recording the fluorescence transients to adjust the detector gain. Subsequently, each leaf sample was exposed to continuous saturating actinic light (3500 μmol photons m^−2^ s^−1^). PAM measurements conducted by the FMS-2 fluorimeter were performed in close proximity to the clips immediately following continuous-excitation chlorophyll fluorescence.

An FMS-2 fluorimeter (Hansatech Instruments Ltd., King’s Lynn, UK) was used for the PAM (pulse-modulated amplitude fluorescence) measurements after adaptation of the plants to light. The following were the 4 measured parameters:

- F_s_—Steady-state fluorescence at any light level. This parameter indicates the intensity of chlorophyll fluorescence, which accompanies the photosynthesis process in stationary conditions.
- F_m’_—Maximum chlorophyll *a* fluorescence in light-adapted leaves.
- Φ_PSII_ (yield or Genty parameter)—Estimated effective quantum yield (efficiency) of PSII photochemistry at a given PAR. Based on changes in the values of this parameter, the quantum yield of the photochemical reaction in PSII can be assessed.
- ETR—electron flow rate through photosystems.

We used the following protocol for PAM measurements:

1. By the use of special leaf clips, plants were adapted to darkness for about 15 min;
2. First pulse light (4000 μmol photons m^−2^ s^−1^) was activated for 1 s (F_o_ and F_m_ measured);
3. Wait until signal gets to a steady state;
4. Actinic light (1000 μmol photons m^−2^ s^−1^) was activated (F_p_ measured);
5. Wait 4–5 min until the signal gets to a steady state (a time that is enough for full stomata adaptation and achieving equilibrium between light and dark phases of photosynthesis) (Fs was measured);
6. Second pulse light (12,000 μmol photons m^−2^ s^−1^) was activated for 1 s (F_m’_ measured).

### 4.5. Statistical Analysis

There were 16 ChFl measurements in each treatment (*n* = 16). Student’s t-test at a 0.05 confidence level was used to analyze the obtained parameters and yield. The mathematical relationship between chlorophyll fluorescence signals, and yield losses were estimated based on Pearson’s correlation coefficient at a 0.05 confidence level. Statistica 10.0 program (Statsoft, Inc., Tulsa, OK, USA) was used to perform all statistical analyses.

## Figures and Tables

**Table 1:** The yield of winter oilseed rape under hail stress (t ha^−1^ ± S.D.). The means marked by the same letter do not differ significantly (*p* < 0.05, *n* = 16).

Treatment	Yield		% Yield Loss
	Mean (t ha^−1^)	±S.D.	
Control	3.84 ^a^	0.09	0.0
Hail	1.77 ^b^	0.07	53.9

**Table 2:** The dependence of gas exchange parameters and chlorophyll content index of the winter oilseed rape after the application hail stress: control. *A_n_*: CO_2_ assimilation (µmol CO_2_ m^−2^ s^−1^); *g_s_*: stomatal conductance (mol H_2_O m^−2^ s^−1^); *E*: transpiration rate (mmol H_2_O m^−2^ s^−1^); *C_i_*: substomatal CO_2_ concentration (µmol CO_2_ mol^−1^); CCI: chlorophyll content index (a.u.). The means marked by the same letter indicate no significant difference (*p* < 0.05, *n* = 16).

Treatment	*A_n_* (µmol CO_2_ m^−2^ s^−1^)		*g_s_* (mol H_2_O m^−2^ s^−1^)		*C_i_* (µmol CO_2_ mol^−1^)		*E* (mmol H_2_O m^−2^ s^−1^)		CCI (a.u.)	
	Mean	±S.D.	Mean	±S.D.	Mean	±S.D.	Mean	±S.D.	Mean	±S.D.
Control	32.2 ^a^	3.0	0.34 ^a^	0.04	238 ^b^	27	17.1	2.1	22.3 ^a^	2.9
Hail	22.5 ^b^	1.7	0.18 ^b^	0.02	351 ^a^	26	8.9	1.4	14.2 ^b^	1.5

**Table 3:** The Pearson correlation coefficient (r) between the yield of winter rapeseed and individual physiological parameters under the influence of hail. Values marked by an asterisk are significant (*p* < 0.05; *n* = 32). The photosynthetic rate in terms of net CO_2_ assimilation (*A_n_*), stomatal conductance (*g_s_*), substomatal CO_2_ concentration (*C_i_*), transpiration rate (*E*), chlorophyll content index (CCI), steady-state fluorescence (F_s_), maximal chlorophyll *a* fluorescence in light-adapted leaves (F_m’_), estimated effective quantum yield (efficiency) of PSII photochemistry at given PAR (Φ_PSII_), electron flow rate (ETR), time to reach the maximal fluorescence (t_Fm_), area above the OJIP curve (Area), minimal fluorescence (F_o_), maximal fluorescence (F_m_), variable fluorescence (F_v_), ratio of the efficiency of primary photochemical reactions (F_o_/F_m_), maximum quantum yield of primary photochemical reactions (F_v_/F_m_), maximum efficiency of the water diffusion reaction (F_v_/F_o_), relative variable fluorescence at the J and I steps (V_j_ and V_i_), normalized total area above the OJIP curve (S_m_), absorption flux per one active RC (ABS/RC), energy flux not intercepted by an RC (DI_o_/RC), energy flux trapped by one active RC (TR_o_/RC), rate of electron transport by one active RC (ET_o_/RC), efficiency index expressed as the density of RCs per chlorophyll (Chl) (RE_o_/RC), maximum quantum yield of primary photochemical reactions (ϕ_Po_), quantum yield for electron transport (ϕ_Eo_), efficiency/probability that an electron moves further than Q_A_^−^ (ψ_Eo_), efficiency/probability with which an electron from the intersystem electron carriers is transferred to reduce end electron acceptors at the PSI acceptor side (δ_Ro_), quantum yield for reduction of end electron acceptors at the PSI acceptor side (ϕ_Ro_), performance indexes (PI_inst_, PI_abs_, and PI_tot_), driving forces (DF_abs_ and DF_tot_).

Parameter	*r*	Parameter	*r*	Parameter	*r*	Parameter	*r*
*A_n_*	0.86 *	Area	−0.02	N	−0.15	ϕ_Ro_	0.05
*g_s_*	0.92 *	F_o_	−0.77 *	ABS/RC	−0.27	PI_abs_	0.41 *
*C_i_*	−0.90 *	F_m_	0.22	DI_o_/RC	−0.11	PI_total_	0.37 *
*E*	0.92 *	F_v_	0.53 *	TR_o_/RC	−0.07	DF_abs_	0.56 *
CCI	0.88 *	F_o_/F_m_	−0.13	ET_o_/RC	−0.52 *	DF_Total_	0.63 *
F_s_	−0.71 *	F_v_/F_m_	0.74 *	REo/RC	−0.03	O	−0.64 *
F_m’_	0.74 *	F_v_/F_o_	0.75 *	ϕ_Po_	0.74 *	K	−0.12
Φ_PSII_	0.94 *	V_j_	0.49 *	ψ_Eo_	−0.49 *	J	0.30
ETR	0.95 *	V_i_	0.02	ϕ_Eo_	−0.29	I	0.05
t _Fm_	0.05	S_m_	−0.14	δ_Ro_	0.18	P	0.14

**Table 4:** Meteorological conditions prevailing in the outside of the greenhouse during the experiment.

	**Average Month Temp. [°C]**		**Min. Month Temp. [°C]**		**Max. Month Temp. [°C]**		**Precipitation [mm]**		**Solar Radiation [W m^−2^]**	
**Month**	**2022**	**2023**	**2022**	**2023**	**2022**	**2023**	**2022**	**2033**	**2022**	**2023**
September	12.6	-	3.2	-	17.2	-	56.0	-	160.3	-
October	11.6	-	0.2	-	16.2	-	31.4	-	132.3	-
November	4.4	-	−6.5	-	6.6	-	22.1	-	48.1	-
December	0.8	-	−13.3	-	2.6	-	61.6	-	29.4	-
January	-	3.6	-	−1.5	-	18.7	-	62.3	-	28.5
February	-	1.8	-	−7.7	-	10.0	-	41.2	-	59.8
March	-	4.9	-	−5.4	-	19.4	-	26.7	-	111.0
April	-	9.4	-	−3.4	-	22.9	-	57.2	-	176.1

**Table 5:** Glossary, definition of terms, and formulae used by the JIP-test for the analysis of the Chl *a* fluorescence transient OJIP emitted by dark-adapted photosynthetic samples ([36]).

t_Fm_	time (in ms) to reach the maximal fluorescence FP (meaningful only when F_P_ = F_m_)
Area	total complementary area between the fluorescence induction curve and F = FP (meaningful only when F_P_ = F_m_)
F_o_ ≅ F50 µs or ≅ F20 µs	fluorescence when all PSII RCs are open (≅ to the minimal reliable recorded fluorescence)
F_m_	maximal fluorescence, when all PSII RCs are closed
F_v_ ≡ F_m_ − F_o_	maximal variable fluorescence
F_v_/F_m_	maximum quantum yield for primary photochemistry
ABS/RC = M_o_ × (1/V_J_) × (1/ϕ_Po_)	absorption flux (exciting PSII antenna Chl *a* molecules) per RC (also used as a unit-less measure of PSII apparent antenna size)
TR_o_/RC = M_o_ × (1/V_J_)	trapped energy flux (leading to Q_A_ reduction), per RC
RE_o_/RC = M_o_ × (1/V_J_) × (1 − V_I_)	electron flux reducing end electron acceptors at the PSI acceptor side, per RC
ET_o_/RC = M_o_ × (1/V_J_) × (1 − V_J_)	electron transport flux (further than Q_A_^−^), per RC
ϕ_Po_ ≡ TR_0_/ABS = [1 − (F_o_/F_m_)]	maximum quantum yield for primary photochemistry
ϕ_Eo_ ≡ ET_o_/ABS = [1 − (F_o_/F_m_)] × (1 − V_J_)	quantum yield for electron transport (ET)
ϕ_Ro_ ≡ RE_o_/ABS = [1 − (F_o_/F_m_)] × (1 − V_I_)	quantum yield for reduction of end electron acceptors at the PSI acceptor side (RE)
ψ_Eo_ ≡ ET_o_/TR_o_ = (1 − V_J_)	efficiency/probability that an electron moves further than Q_A_^−^
δ_Ro_ ≡ RE_o_/ET_o_ = (1 − V_I_)/(1 − V_J_)	efficiency/probability with which an electron from the intersystem electron carriers is transferred to reduce end electron acceptors at the PSI acceptor side (RE)
N = S_m_ × (M_o_/V_J_)	turnover number (expresses how many times Q_A_ is reduced in the time interval from 0 to t_FM_)
PI_abs_	performance index for energy conservation from photons absorbed by PSII until the reduction of intersystem electron acceptors
PI_total_	total performance index for energy conservation from photons absorbed by PSII until the reduction of PSI end electron acceptors

## Data Availability

The data published in this article are available from the corresponding author.
